# Inhibition of N-linked glycosylation impairs ALK phosphorylation and disrupts pro-survival signaling in neuroblastoma cell lines

**DOI:** 10.1186/1471-2407-11-525

**Published:** 2011-12-22

**Authors:** Federica Del Grosso, Marilena De Mariano, Lorena Passoni, Roberto Luksch, Gian Paolo Tonini, Luca Longo

**Affiliations:** 1 Translational Oncopathology, IRCSS A.O.U. San Martino-IST, National Cancer Research Institute, L.go R. Benzi 10, 16132 Genoa, Italy; 2 Department of Experimental Medicine, University of Genoa, Via L. Battista Alberti 2, 16132 Genoa, Italy; 3 Pediatric Oncology Unit, Fondazione IRCCS Istituto Nazionale dei Tumori, Via Venezian 1, 20133 Milano, Italy; 4 Italian Neuroblastoma Foundation, L.go Gaslini 5, 16147 Genoa, Italy; 5 Department of Medical Pharmacology, University of Milano, Via Vanvitelli 32, 20129 Milano, Italy

## Abstract

**Background:**

The Anaplastic Lymphoma Kinase (ALK) is an orphan receptor tyrosine kinase, which undergoes post-translational N-linked glycosylation. The catalytic domain of ALK was originally identified in the t(2;5) translocation that produces the unglycosylated oncogenic protein NPM-ALK, which occurs in Anaplastic Large Cell Lymphoma (ALCL). Recently, both germline and somatic activating missense mutations of ALK have been identified in neuroblastoma (NB), a pediatric cancer arising from neural crest cells. Moreover, we previously reported that ALK expression is significantly upregulated in advanced/metastatic NB. We hypothesized that ALK function may depend on N-linked glycosylation and that disruption of this post-translational modification would impair ALK activation, regardless the presence of either gene mutations or overexpression.

**Methods:**

We employed tunicamycin to inhibit N-linked glycosylation. The following ALK-positive NB cell lines were used: SH-SY5Y and KELLY (ALK mutation F1174L), UKF-NB3 (ALK mutation R1275Q) and NB1 (ALK amplification). As a control, we used the NB cell lines LA1-5S and NB5 (no ALK expression), and the ALCL cell line SU-DHL1 (NPM-ALK).

**Results:**

Tunicamycin treatment of ALK-positive NB cells resulted in a hypoglycosylated ALK band and in decreased amounts of mature full size receptor. Concomitantly, we observed a marked reduction of mature ALK phosphorylation. On the contrary, tunicamycin had no effects on NPM-ALK phosphorylation in SU-DHL1 cells. Moreover, phosphorylation levels of ALK downstream effectors (AKT, ERK1/2, STAT3) were clearly impaired only in ALK mutated/amplified NB cell lines, whereas no significant reduction was observed in both ALK-negative and NPM-ALK-positive cell lines. Furthermore, inhibition of N-linked glycosylation considerably impaired cell viability only of ALK mutated/amplified NB cells. Finally, the cleavage of the Poly-ADP-ribose-polymerase (PARP) suggested that apoptotic pathways may be involved in cell death.

**Conclusions:**

In this study we showed that inhibition of N-linked glycosylation affects ALK phosphorylation and disrupts downstream pro-survival signaling, indicating that inhibition of this post-translational modification may be a promising therapeutic approach. However, as tunicamycin is not a likely candidate for clinical use other approaches to alter N-linked glycosylation need to be explored. Future studies will assess whether the efficacy in inhibiting ALK activity might be enhanced by the combination of ALK specific small molecule and N-linked glycosylation inhibitors.

## Background

Neuroblastoma (NB) is a pediatric cancer that arises from neural crest cells committed to the adrenal medulla and the sympathetic nervous system. NB accounts for 7-10% of all childhood cancers and the disease prevalence is about one case in 7,000 live births [1]. Most NB cases are sporadic and may occur either as a localized disease with a favorable prognosis or as a disseminated disease, which, on the contrary, has a worse prognosis in children over 1 year of age and heavily contributes to childhood cancer mortality [1]. In 2008, we participated in discovering that activating germline missense mutations in the catalytic domain of the Anaplastic Lymphoma Kinase (ALK) are a major cause for predisposition to familial NB [2]. In addition, ALK mutations were also found to be somatically acquired in about 8% of sporadic NB cases, suggesting that ALK is involved in NB carcinogenesis in at least a subset of tumors [2-8]. Moreover, we have investigated ALK expression levels in relationship with the presence of ALK mutations and the clinical outcome of patients, observing that ALK protein expression is significantly upregulated in advanced/metastatic NB, regardless the presence of gene mutations [7].

ALK is an orphan trans-membrane receptor tyrosine kinase (RTK), which belongs to the insulin receptor superfamily of RTKs. The catalytic domain of ALK was originally identified in the t(2;5)(p23;q35) chromosomal translocation that produces the unglycosylated oncogenic fusion protein NPM-ALK, which occurs in Anaplastic Large Cell Lymphoma (ALCL) [9]. ALK is a protein of approximately 180 kDa that consists of 1,620 amino acids and undergoes post-translational modifications such as glycosylation of asparagines, namely, N-linked glycosylation. Indeed, ALK has 16 highly conserved putative sites of N-linked glycosylation in the extra-cellular portion. Consequently, the mature full-length receptor has a molecular weight of about 220 kDa.

N-linked glycosylation is a highly regulated post-translational modification, which is involved in several biological processes such as protein folding and conformation, oligomerization, sorting, cell-cell interactions, and targeting of proteins to sub- or extra-cellular locations [10,11]. Moreover, several reports have shown a crucial role of N-linked carbohydrates in cell-cycle progression and cell viability [11]. This post-translational modification is initiated upon entry of the polypeptide into the lumen of the endoplasmic reticulum and involves transfer of a carbohydrate moiety to an asparagine (N) residue within a specific amino acid consensus sequence (i.e. NXS/T). The carbohydrate side chain is then processed in the endoplasmic reticulum and Golgi network to produce a mature glycoprotein that is exported through the secretory machinery to the plasma membrane.

Tunicamycin is a specific inhibitor of N-linked glycosylation that blocks the first step of glycoprotein synthesis, thus inhibiting the synthesis of all N-linked glycoproteins. Interestingly, tunicamycin was shown to impair the function of several RTKs such as EGFR, ErbB2, ErbB3, and IGF-IR [12].

We hypothesized that, as demonstrated for other RTKs [12], ALK function may depend on N-linked glycosylation and we investigated the effects of tunicamycin on NB cell lines characterized by different ALK alterations. Our results showed that inhibition of N-linked glycosylation impairs ALK phosphorylation and disrupts downstream pro-survival signaling, as well as cell viability, in NB lines in which ALK is mutated or amplified.

## Methods

### Cell culture

The following ALK-positive NB cell lines were employed: SH-SY5Y and KELLY (F1174L ALK mutation), UKF-NB3 (R1275Q ALK mutation) and NB1 (ALK amplified). As a control, we included LA1-5S and NB5 NB cell lines that are both ALK-negative, and the ALCL derived cell line SU-DHL1, which harbors the fusion protein NPM-ALK. Human NB cell line SH-SY5Y was purchased from the Biological Bank and Cell Factory core facility (National Cancer Research Institute, Genoa, Italy) and the LA1-5S cell line was purchased from the European Collection of Cell Cultures (ECACC). UKF-NB3, NB1, NB5, KELLY and SU-DHL1 cell lines were kindly provided by the Pediatric Oncology Unit (Fondazione IRCCS Istituto Nazionale dei Tumori, Milan, Italy). All cell lines employed in this study have been rechecked for ALK mutations, alterations and expression levels of the protein. LA1-5S were grown in MEM/Ham's F12 1:1 supplemented with non-essential amino acids and 10% inactivated fetal bovine (Lonza, Basel, Switzerland). All other cell lines were grown in RPMI 1,640 supplemented with 10% inactivated fetal bovine serum (Lonza). Tunicamycin was purchased from Sigma-Aldrich (St. Louis, MO).

### Western blot analysis

A standard immunoblotting protocol was used to assay protein expression in cell lysates. Cells were washed twice with ice-cold PBS and lysed with 100-200 μl of lysis buffer (Invitrogen, Carlsbad, CA), supplemented with protease and phosphatase inhibitor cocktails and PMSF (phenylmethylsulfonyl fluoride) (Sigma-Aldrich). Lysates were vortexed and incubated on ice for 10 min twice and then cleared by spinning at 20,000 × g for 10 min at 4°C. Proteins were separated by 7.5%-10% SDS-PAGE gels and immunoblotted according to standard Western blotting procedures using primary antibodies for ALK, AKT, ERK1/2, STAT3 and related phosphoproteins (Cell Signaling, Danvers, MA), and either α-tubulin (Cell Signaling) or GAPDH (Abcam, Cambridge, UK) as a control for loading of equal amounts of cell lysates. Goat anti-rabbit and goat anti-mouse IgG HRP conjugated secondary antibodies were from Jackson Immunoresearch Inc (Suffolk, UK) and Santa Cruz Biotechnologies Inc (Santa Cruz, CA), respectively. Blots were developed with Amersham's ECL (GE Healthcare, Waukesha, WI).

Quantification of the western blot bands was performed by ImageJ [13].

### MTT assay

Six replicates of SH-SY5Y, KELLY, UKF-NB3, NB1 and SU-DHL1 (5,000 cells/well) and of LA1-5S (2,500 cells/well) were plated in 96-well plates with 100 μl RPMI or MEM + Ham's F12 medium additioned with 10% fetal bovine serum. After 24 h cells were treated with decreasing concentrations of tunicamycin (500 nM - 200 nM - 100 nM - 50 nM - 25 nM), whereas no treatment was done as positive proliferation control. At 48 h, 25 μl of 2 mg/ml MTT (Sigma-Aldrich) was added and plates incubated for 4 h at 37°C and centrifuged 10 min at 1,500 rpm. Finally, 150 μl of DMSO were added to each well and absorbance was read at 560 nm.

### FACS analysis

Ethanol fixed cells were incubated with 5 μl annexin-V FITC conjugate (Invitrogen) for 10 min at room temperature in the dark. Thereafter, cells were centrifuged, resuspended in 190 μl binding buffer and additioned with 10 μl propidium iodide (PI) (Sigma-Aldrich). Cells were analyzed immediately for Annexin-V-FITC and PI binding, using a FACS (fluorescence activated cell sorting) flow cytometer (CyAn™ Dako). Dot plots and histograms were analyzed by the Dako CyAn - Summit 4.3 software. Annexin-V and PI positive cells were considered apoptotic. Annexin-V and PI negative cells were considered viable.

### xCELLigence real-time cell analysis

SH-SY5Y, KELLY, NB1, LA1-5S (5,000 cells/well), and UKF-NB3 (40,000 cells/well) cells were seeded in E-Plates 16 (Roche, Basel, CH) in appropriate culture media to a final volume of 200 μl and maintained in a CO_2 _incubator at 37°C and 5% CO_2 _saturation. Dynamic cell proliferation was monitored in real-time from the time of plating until the end of the experiment. Electrode impedance at the bottom of each well was displayed as cell index, which is a direct measure of cell adhesion and it is a measure that indicates a change in cell behavior due to an increase or decrease in cell number, cell adhesion or cell morphology. Adherent NB cell lines were firstly tested to determine the optimal number of cells to be plated, and cells were allowed to reach a cell index of at least 0.5-1 before treatment. Exponentially growing cells were treated in duplicate with decreasing concentrations of tunicamycin (500 nM, 250 nM and 125 nM). No treatment was used as positive proliferation control. Cell index values were acquired in real-time for at least 48 h after treatment. Cell index curves were calculated for every tunicamycin treatment and plotted on a graph by Excel.

## Results

### Inhibition of N-linked glycosylation impairs ALK phosphorylation and downstream signaling

To investigate the effect that inhibition of N-linked glycosylation could have on ALK phosphorylation and downstream signaling we treated cells with 500 nM tunicamycin, a concentration previously employed in other cell lines of different origin [12], in time course experiments.

**Figure 1:**
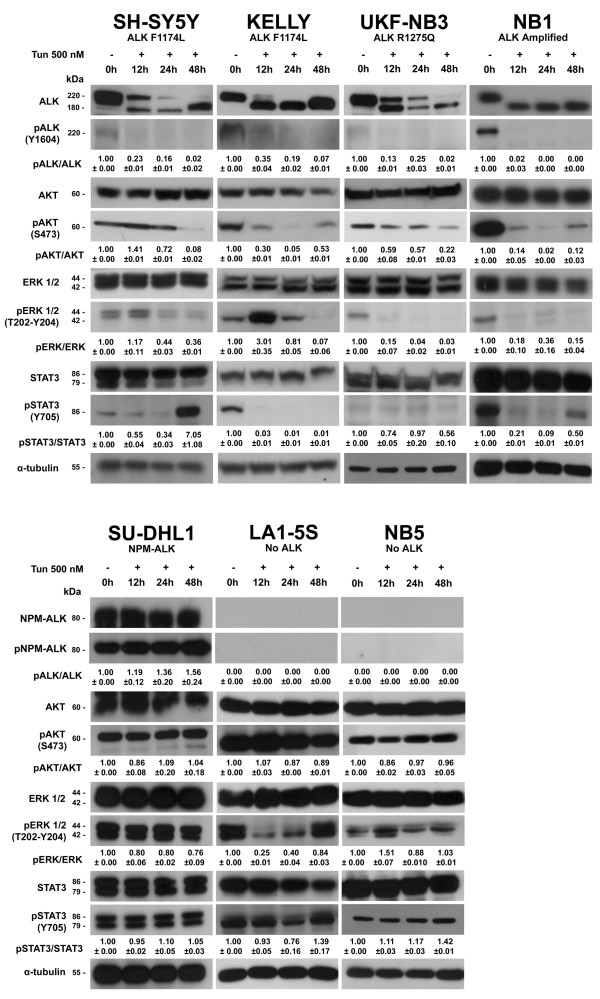
**Effects of tunicamycin on ALK expression and phosphorylation and on downstream signaling proteins**. Cells were exposed to 500 nM tunicamycin and treated in time course experiments. Expression levels of ALK and phosphorylation of ALK (Tyr1604), AKT (Ser473), ERK1/2 (Thr202/Tyr204), STAT3 (Tyr705) and NPM-ALK (Tyr664) were detected in total cell lysates by Western blot, using corresponding antibodies. α-tubulin was used as sample loading control. Quantification of western blot bands was performed by using ImageJ software [13]. Quantification values for each band were firstly normalized to corresponding values of α-tubulin. Then, normalized values for each time were compared to the corresponding band of the untreated sample (0 hours). Finally the ratio between the phosphorylated form of the proteins and its unphosphorylated counterpart for each experimental time was calculated and reported with standard deviations.

Western blot analysis of protein levels showed the accumulation of a hypoglycosylated ALK band (180 kDa) and a concomitant decreased of the amount of mature full size receptor (220 kDa) (Figure 1). Interestingly, after 12 h of treatment, we also observed a marked decrease of ALK phosphorylation in each of the NB cell lines harboring ALK mutation or amplification, and the unphosphorylated status of ALK was still present at 48 h (Figure 1). On the contrary, tunicamycin treatment performed on the ALCL derived cell line SU-DHL1 did not result in any reduction of the phosphorylation levels of NPM-ALK, which lacks N-linked glycosylation consensus sites.

Next, we investigated expression and phosphorylation levels of proteins involved in ALK signaling (i.e. AKT, ERK1/2 and STAT3). Tunicamycin treatment did not affect AKT, ERK1/2 and STAT3 expression (Figure 1), whereas their phosphorylation levels decreased considerably within 48 h. Noteworthy, we detected an evident rephosphorylation of STAT3 in SH-SY5Y cells after 48 h of tunicamycin treatment (Figure 1). As tunicamycin impairs N-linked glycosylation of all newly synthesized glycoproteins, we also treated LA1-5S, NB5 and SU-DHL1 cell lines, which do not rely on native ALK as an oncogenic driver. Intriguingly, tunicamycin did not affect phosphorylation levels of ALK downstream effectors in these control cell lines, apart from a temporary reduction of phospho-ERK1/2 levels in LA1-5S cells (Figure 1).

### Inhibition of N-linked glycosylation affects cell viability of ALK-driven NB cell lines

**Figure 2:**
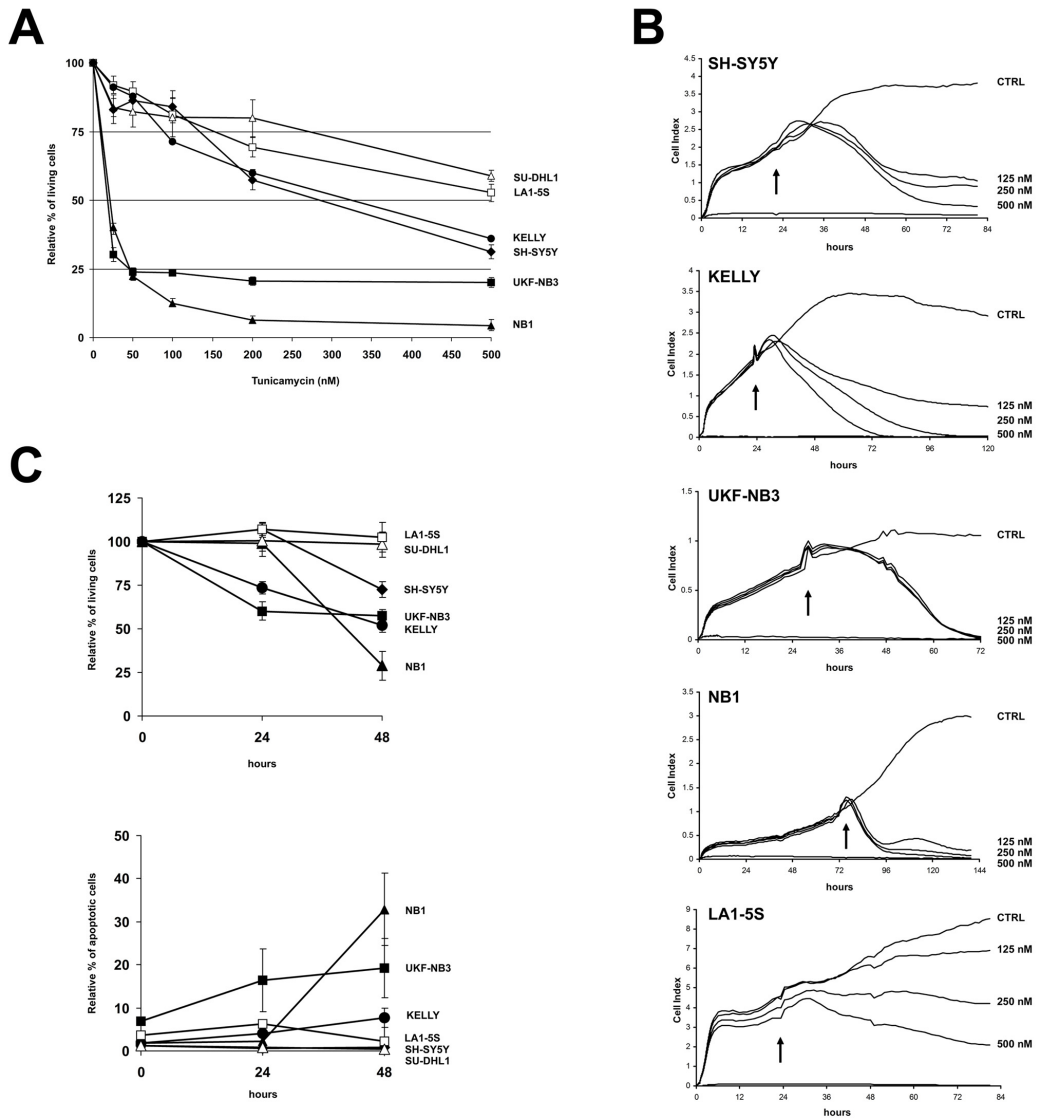
**Effects of tunicamycin on cell viability**. (A) Cell lines were exposed to varying concentrations of tunicamycin for 48 h and cell survival fractions were measured by MTT assay. (B) Cell lines were exposed to decreasing concentrations of tunicamycin and cell viability monitored by the xCELLigence™ instrument. The cell index achieved by this technology is a measurement derived from electrical impedance of cells interacting with microelectrodes on the bottom of a 16-well plate. Cell growth was assessed in real-time for at least 48 h after tunicamycin administration to cell medium. Arrows indicate time of treatment; CTRL: untreated control. (C) Cells were exposed to 500 nM tunicamycin for 24 and 48 h and analyzed by FACS. The upper plot shows the relative percentage of viable cells, which were double negative for both Annexin-V and PI. The lower plot shows Annexin-V and PI positive cells, which were considered apoptotic.

To evaluate the effect of inhibition of N-linked glycosylation on cell survival, cells were cultured for 48 h with increasing concentrations of tunicamycin, ranging from 25 nM to 500 nM, and cell viability was tested by MTT assay (Figure 2A). Cell survival fractions were evaluated in comparison to control cell survival. We observed a profound inhibition of cell growth in both UKF-NB3 and NB1 cells that showed an IC_50 _of about 20 nM, whereas SH-SY5Y and KELLY cells were more resistant, showing an IC_50 _of 283 nM and 325 nM, respectively (Figure 2A). On the other hand, both ALK-negative LA1-5S cells and NPM-ALK-positive SU-DHL1 cells were definitely resistant to tunicamycin, showing an IC_50 _> 500 nM (Figure 2A).

**Figure 3:**
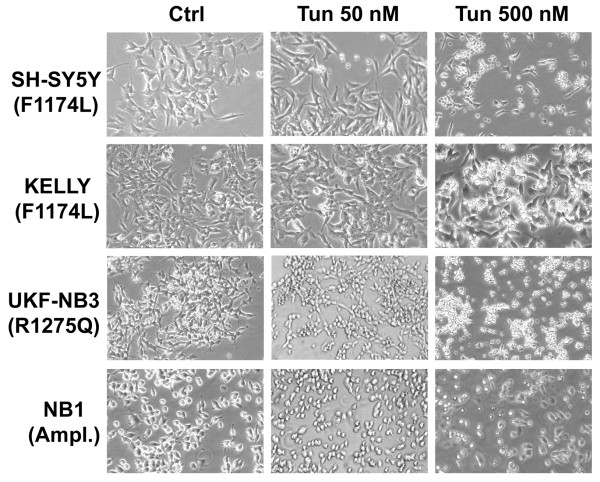
**Photomicrographs of ALK-positive NB cell lines**. ALK-positive NB cell lines were treated with tunicamycin at the indicated concentrations for 48 h. At a tunicamycin concentration of 500 nM all cell lines looked suffering and most of the cells were detached.

Cell viability was also analyzed by xCELLigence™ instrument (Roche), which monitors cell adhesion in real-time (Figure 2B). As a result, cell index of each NB cell lines harboring ALK mutation or amplification was extensively impaired, whereas cell index of LA1-5S cells resulted minimally affected (Figure 2B). Noteworthy, in contrast to MTT results, we observed a marked decrease of cell growth also for SH-SY5Y and KELLY cells. In fact, as for the other ALK-positive cell lines, also SH-SY5Y and KELLY looked suffering at observation and most of the cells were detached from the bottom of the flask after 48 h treatment with 500 nM tunicamycin (Figure 3).

Finally, cell lines were analyzed by flow cytometry after double staining with annexin-V and PI. After 48 h of treatment with 500 nM tunicamycin, SH-SY5Y, KELLY, UKF-NB3, and NB1 cells showed a cell viability reduction of 27%, 48%, 42%, and 71%, respectively, whereas no significant variation was observed in both LA1-5S and SU-DHL1 cells (Figure 2C). Moreover, about 6% of KELLY, 13% of UKF-NB3 and 31% of NB1 cells displayed double staining to both annexin-V and PI, and were considered apoptotic (Figure 2C).

**Figure 4:**
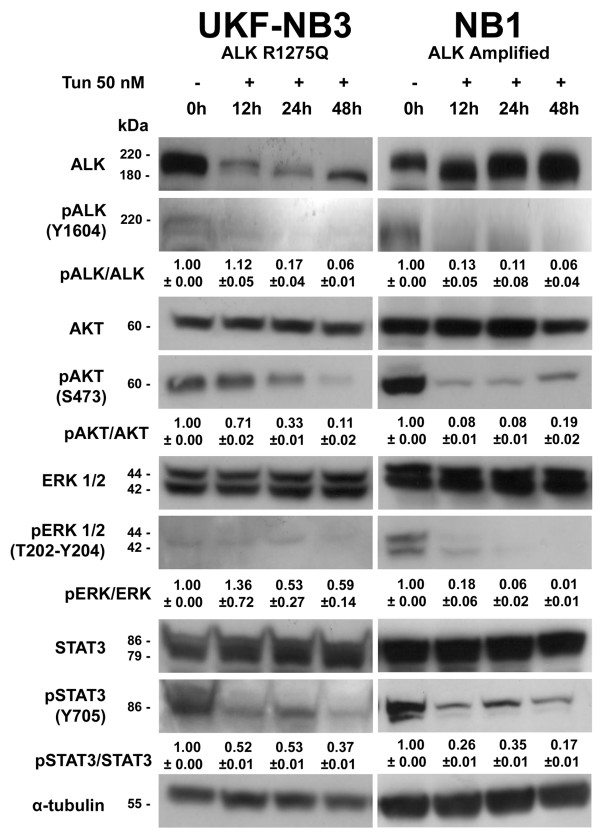
**Effects of tunicamycin 50 nM on NB1 and UKF-NB3 cells**. In both NB cell lines that showed a lower IC_50 _by MTT assay (NB1 and UKF-NB3) we observed similar effects to those detected by using 500 nM tunicamycin. Quantification of western blot bands was performed by using ImageJ software [13] as described in Figure 1.

Since both NB1 and UKF-NB3 showed an IC_50 _greatly lower than the tunicamycin concentration used in the first set of experiments, we employed 50 nM tunicamycin in ALK-positive NB cell lines. As a result, we observed similar effects on ALK and its effectors in NB1 and UKF-NB3 (Figure 4) but not in SH-SY5Y and KELLY cells, which in fact were more resistant as detected by MTT assay.

### Activation of apoptotic pathways in tunicamycin treated cells

**Figure 5:**
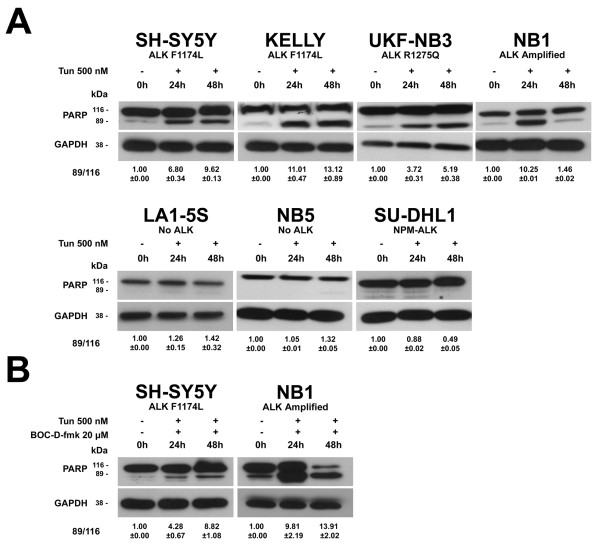
**Activation of the Poly-ADP-ribose-polymerase (PARP) protein**. Cells were treated for 48 h with: (A) 500 nM tunicamycin only; (B) 500 nM tunicamycin and 20 μM of the pan-caspase inhibitor BOC-D-fmk. GAPDH was used as sample loading control. Quantification of western blot bands was performed by using ImageJ software [13] as described in Figure 1, except the ratio was calculated between the cleaved and the uncleaved form of PARP.

To investigate the involvement of apoptotic pathways after inhibition of N-linked glycosylation we analyzed the following apoptotic proteins: caspase-3, caspase-8, caspase-9, caspase-12 and the Poly-ADP-ribose-polymerase (PARP). Cells were exposed to 500 nM tunicamycin for 48 h. We did not detect cleavage fragments in any of the analyzed caspases. On the contrary, we observed cleavage fragments of PARP in all ALK-positive NB cell lines but not in control cell lines (Figure 5A). Cleavage fragments of PARP were still present after treatment of SH-SY5Y and NB1 cells with both 20 μM of the pan-caspase inhibitor BOC-D-fmk (Calbiochem, Gibbstown, NJ) and 500 nM of tunicamycin, indicating a caspase independent activation of PARP (Figure 5B).

## Discussion

In the present study, we addressed the question of whether ALK function may depend on N-linked glycosylation. Inhibition of ALK activity in NB cell lines has already been approached by using specific small molecule ALK inhibitors, such as PF-2341066 [3], NVP-TAE684 [4] and CEP14083/CEP14513 [7], and more recently by RNA interference molecules [14]. Particularly, NB cells harboring either R1275Q mutation or ALK amplification showed sensitivity to PF-02341066 and these results were also confirmed in xenografts (Wood AC et al., ASCO Annual Meeting, 2009). However, NB cell lines with both F1174L ALK mutations and wild type ALK were more resistant. Furthermore, two secondary mutations in the kinase domain of the fusion protein EML4-ALK were discovered in non-small-cell lung cancer in tumor cells isolated from a patient during the relapse phase of treatment with the ALK inhibitor PF-02341066 [15]. Each mutation developed independently in subclones of the tumor and conferred marked resistance to two different ALK inhibitors, PF-02341066 and a 2,4-pyrimidinediamine derivative (PDD) [15]. Although small molecule inhibitors are a class of anticancer compounds that have shown promising clinical activity, resistance phenomena may occur, in part due to either primary and secondary mutations or to the plasticity of regulation of downstream signaling pathways. Hence, great efforts are now aimed at finding efficient ALK inhibitors, at least for the more frequent ALK alterations.

In 2008, Contessa and colleagues reported that inhibition of N-linked glycosylation reduced RTK (i.e. EGFR, ErbB2, ErbB3 and IGF-IR) signaling through AKT and radiosensitized tumor cells in glioma and pancreatic adenocarcinoma cell lines. In comparison, experiments carried out in nontransformed fibroblastic cells showed neither a reduction in RTK dependent signaling nor an enhancement in radiosensitivity [12]. Afterwards, they also provided evidences that disruption of N-linked glycosylation could reduce RTK signaling *in vivo *and increase radiosensitivity of gliomas, suggesting that targeting N-linked glycosylation may combine favorably with other classes of EGFR inhibitors to reduce both oncogenic signaling and the mechanisms of therapeutic resistance [16]. Moreover, tunicamycin was showed to enhance the susceptibility of lung cancer cells and sensitize resistant cell lines to Erlotinib [17].

Similarly, our results indicated that N-linked glycosylation has a pivotal role also in ALK activation, as described for EGFR and other RTKs [12]. Tunicamycin has broad, non-specific effects and acts on all N-linked glycoproteins. Nevertheless, we specifically investigated the effects of tunicamycin on NB cell lines that are dependent on ALK for survival. Here, we showed that inhibition of N-linked glycosylation led to reduced or undetectable phosphorylation of ALK. Moreover, we also observed that pro-survival ALK effectors are inactivated only in NB cell lines addicted to native ALK receptor as an oncogenic driver. On the contrary, ALK-negative NB cell lines did not show evident impairment of signaling molecules such as AKT, ERK1/2 and STAT3. Noteworthy, although tunicamycin has broad effects, we did not observe any variation in the phosphorylation levels of ALK effectors in SU-DHL1 cells, which depend on the unglycosylated cytoplasmatic protein NPM-ALK. The reactivation of STAT3 observed in SH-SY5Y cells may be explained by considering a possible redundancy of cellular signaling networks. Indeed, multiple RTKs may be coactivated in tumors and redundant inputs drive and maintain downstream signaling [18,19], although other possibilities should not be ruled out.

Inhibition of N-linked glycosylation also impaired cell proliferation only of ALK-positive NB cells. Particularly, UKF-NB3 and NB1 cells showed high sensitivity to tunicamycin by MTT assay, whereas SH-SY5Y and KELLY cells were less sensitive. However, cell viability assays carried out by flow cytometry and real-time monitoring of cell adhesion showed a marked cell growth reduction for all ALK-positive NB cell lines, including SH-SY5Y and KELLY. Finally, the cleavage of the Poly-ADP-ribose-polymerase (PARP) was observed only in ALK-positive NB cell lines, suggesting that apoptotic pathways may be involved in cell death.

## Conclusions

In this study we showed that the inhibition of N-linked glycosylation has the capability to affect ALK phosphorylation and disrupt pro-survival signaling associated to ALK, indicating that inhibition of this post-translational modification may be a promising therapeutic approach for ALK-depending NB patients. Moreover, the contributions of coexpressed growth factor receptors to developing resistance to therapeutic RTK inhibitors have been shown, confirming that inhibition of multiple RTKs may be of therapeutic benefit. Thus, as tunicamycin acts on all N-linked glycoproteins it is plausible to suppose that, differently from specific small molecule inhibitors, disruption of N-linked glycosylation should reduce a reliance on networks of RTKs that may overlap in effects with ALK.

Since tunicamycin is not a likely candidate for clinical use our results are to be taken as a proof-of-concept and other approaches to alter N-linked glycosylation need to be explored. Future studies will assess whether the efficacy in inhibiting ALK activity might be enhanced by the combination of ALK specific small molecule and N-linked glycosylation inhibitors.

## Competing interests

The authors declare that they have no competing interests.

## Authors' contributions

FDG designed and performed experiments, interpreted results. MDM performed experiments. LP performed experiments and critically revised the manuscript. RL critically revised the manuscript. GPT critically revised the manuscript. LL conceived of the study, designed experiments, interpreted results, drafted and critically revised the manuscript. All authors read and approved the final manuscript.

## Pre-publication history

The pre-publication history for this paper can be accessed here:

http://www.biomedcentral.com/1471-2407/11/525/prepub
